# Severe disseminated clinical presentation of monkeypox virus infection in an immunosuppressed patient: first death report in Brazil

**DOI:** 10.1590/0037-8682-0392-2022

**Published:** 2022-08-29

**Authors:** Yargos Rodrigues Menezes, Alexandre Braga de Miranda

**Affiliations:** 1 Fundação Hospitalar do Estado de Minas Gerais, Hospital Eduardo de Menezes, Programa de Residência Médica em Dermatologia, Belo Horizonte, MG, Brasil.; 2 Fundação Hospitalar do Estado de Minas Gerais, Hospital Eduardo de Menezes, Programa de Residência Médica em Infectologia, Belo Horizonte, MG, Brasil.

**Keywords:** Monkeypox virus, HIV, Death

## Abstract

Since May 2022, the number of monkeypox virus infections has sharply increased in countries where the disease has not been previously endemic. At present, most reports refer to low-severity cases. Herein, we present a severe case of the disease with disseminated skin lesions that progressed to death in an immunosuppressed patient in Belo Horizonte, Minas Gerais, Brazil.

## INTRODUCTION

Monkeypox virus was first identified in monkeys in 1957 at the Statens Serum Institut in Copenhagen, Denmark. The first case of human infection was reported in the Democratic Republic of the Congo in 1970. Since then, sporadic outbreaks have been reported outside the African continent^1^.

However, since May 2022, the number of infections has sharply increased in several countries where the disease was not endemic. The disease is transmitted through direct contact with lesions containing the virus. In the current outbreak, most cases were reported in people who had not traveled to countries where the disease is endemic. The majority of the cases were from males who had sex with males (MSM), which reinforces the possibility of sexual transmission. Although monkeypox is not considered to be a sexually transmitted disease, the virus can be inoculated into the skin and mucosa through sexual intercourse and intimate contact^1^.

Herein, we report the case of a patient in Belo Horizonte, Minas Gerais, Brazil, with a confirmed monkeypox virus infection with a severely disseminated clinical presentation, which differs from the description of existing cases in the current outbreak worldwide. To date, this case report represents the first death caused by this disease outside of Africa during the current outbreak.

## CASE REPORT

A 41-year-old male, born in Pará de Minas, Minas Gerais, Brazil, and living in Belo Horizonte, noticed the onset of papulovesicular lesions with central umbilication on July 7, 2022. In addition, he reported painful bilateral lymphadenopathy in the inguinal region. On July 9, 2022, the patient developed diarrhea, weakness, and malaise. The patient did not have a fever, flu prodrome, or other symptoms. The number of lesions in the integument increased rapidly, and on July 14, 2022, the patient was referred to the Hospital Eduardo de Menezes, a specialized hospital for the treatment of infectious contagious diseases in Belo Horizonte, Minas Gerais, Brazil.

The first skin lesions appeared on the forehead and rapidly progressed to the rest of the body, including the chest, abdomen, back, upper and lower limbs, palms of the hands, soles of the feet, genitalia, perineum, anorectal region, tongue, and oropharynx. The patient had no history of traveling abroad or to other Brazilian states; however, he reported unprotected sexual contact on June 30, 2022, with a male who had traveled to the countryside of the state of Minas Gerais a few days earlier.

The patient was an MSM, carrier of the human immunodeficiency virus (HIV), diagnosed in 2005, and had been undergoing regular treatment since 2021. His viral load was undetectable and the CD4 lymphocyte count was 53 on May 31, 2022. Previously, in November 2021, his viral load was undetectable and the CD4 lymphocyte count was 74. 

He reported his last chemotherapy cycle on July 5, 2022, for the treatment of diffuse large B-cell lymphoma with metastases to the spine, skull, and liver. The first chemotherapy cycle was initiated in February 2022. Laboratory review at hospital admission showed anemia (hemoglobin = 8.1 g/dL) and a global leukocyte count of 500 cells/mm³ (35 neutrophils/mm³), without other abnormalities. Chest radiography revealed no pathological findings.

**FIGURE 1:**
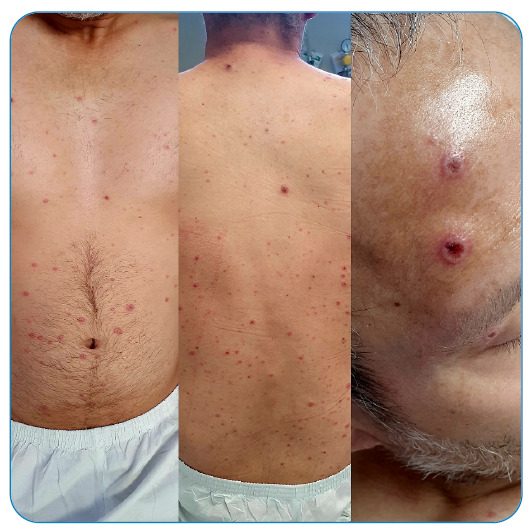
Hospital admission on July 15, 2022.

**FIGURE 2:**
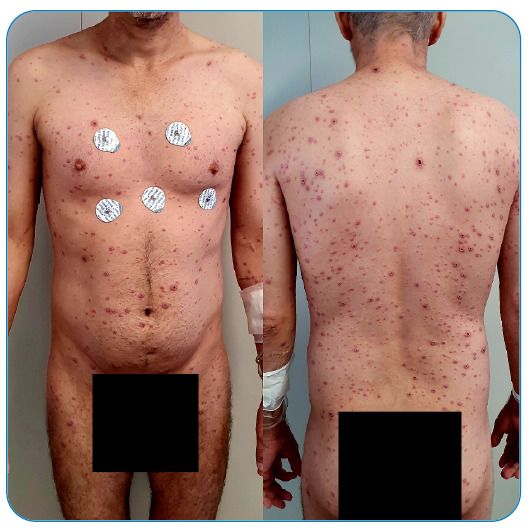
July 18, 2022.

**FIGURE 3:**
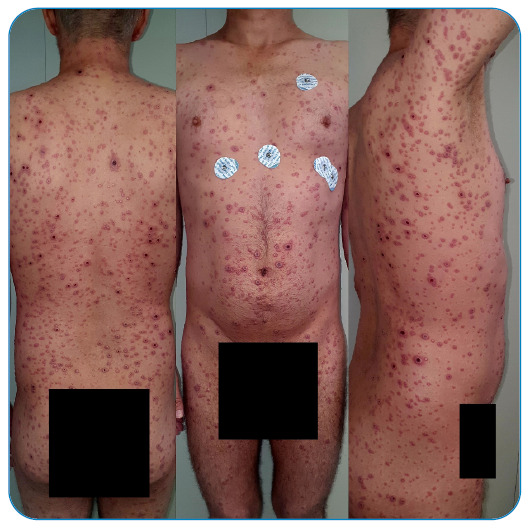
July 21, 2022.

**FIGURE 4:**
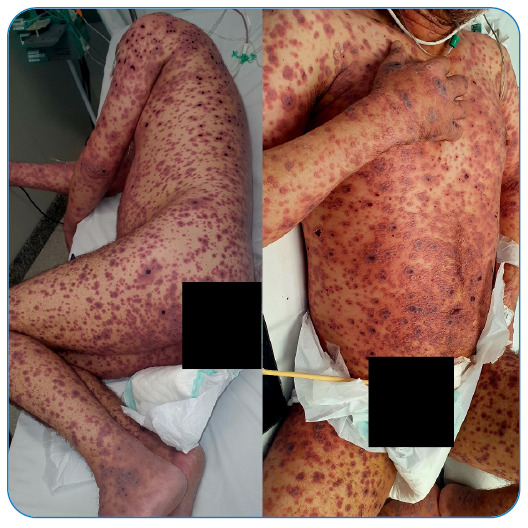
July 27, 2022.

**FIGURE 5:**
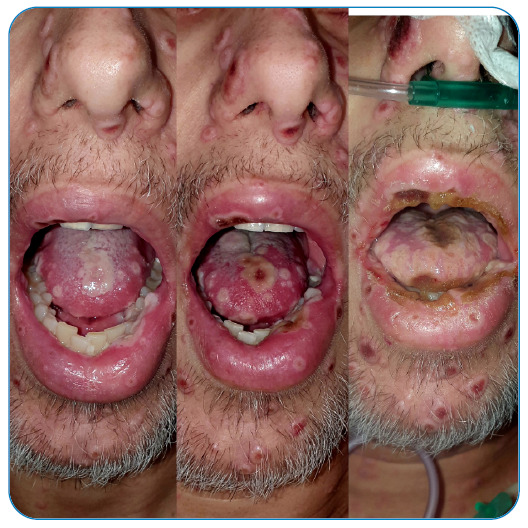
Oral cavity lesions.

The liquid contained in the cutaneous vesicles was collected on July 15, 2022, for RT-PCR, which was positive for monkeypox virus. Molecular diagnosis was performed at the Fundação Ezequiel Dias (FUNED) in Belo Horizonte. The number of lesions progressively increased from the onset of the first lesion until July 25, 2022, making it a period of approximately two and a half weeks (Figures 1, 2, 3, and 4). The many lesions in the oral cavity caused pharyngitis, limiting oral intake (Figure 5). Therefore, the patient required a nasoenteric tube for feeding. 

Concomitantly, with the appearance of skin lesions on July 23, 2022, the patient progressively developed dyspnea, requiring supplemental oxygen via a nasal catheter, suggesting a possible pulmonary complication of the virus. Meropenem and vancomycin were prescribed empirically on the same day. On July 26, 2022, the patient underwent a chest radiograph showing bilateral diffuse interstitial infiltrates. Unfortunately, it was not possible to perform a chest computerized tomography (CT) scan because of the absence of equipment at the hospital.

On July 24, 2022, the onset of significant edema was observed on the penis and glans, causing an anatomical deformation of the region and urinary flow obstruction, requiring cystostomy on July 25, 2022.

On July 27, 2022, the patient suddenly developed significant respiratory deterioration, acute kidney injury, and multiple organ dysfunctions. Therefore, he was referred to the intensive care unit (ICU) where he died on July 28, 2022. There was no bacterial growth in several blood cultures and the patient was under a broad-spectrum antibiotic therapy. 

## DISCUSSION

Guarner et al. reported an incubation period of 5 to 21 days, which is consistent with that observed in our patient, who had an incubation period of 7 days. In addition to skin lesions, other symptoms may be present, such as fever, headache, myalgia, fatigue, and lymphadenopathy. Skin lesions consist of macules and papules that progress to vesicles, ulcers, and crusts^1^. In our patient, crusts were more significant two weeks after the appearance of the first skin lesion.

Skin lesions usually start near inoculation sites, which may explain the fact that the clinical presentation of skin lesions is mostly localized near the genitalia and anus in the current outbreak^1^ ^,^ ^2^. However, our patient presented with disseminated lesions throughout the body and reported an onset of the first lesion in the forehead region. The lesions were all in the same clinical stage throughout the course of the disease, which was also observed by Perez et al^3^. Although the clinical course of the disease is well established in the medical literature, lasting from 2 to 4 weeks, there is no mention of the period between the onset of the first and last skin lesions. The progressively increasing number of new lesions presented by the patient lasted approximately two and a half weeks, when most of them were in the crusted stage.

The infection is generally limited. However, complications, such as encephalitis, pneumonia, and secondary skin lesions, may occur. Children, pregnant women, and immunosuppressed individuals are more likely to develop complications and severe disease^1^ ^,^ ^3^ ^,^ ^4^. The patient had a history of chemotherapy for lymphoma, and the last cycle of chemotherapy occurred five days after the probable date of contamination. The serious immunological impairment caused by chemotherapy may explain the observed severe disseminated clinical presentation. 

There is no specific treatment for monkeypox, and therapy is mostly symptomatic. Nevertheless, there are currently two antiviral drugs that may be used for monkeypox in patients with severe disease: tecovirimat and brincidofovir. Neither tecovirimat nor brincidofovir is currently available for use in Brazil^1^.

Thornhill et al. found in a study of 528 cases in 16 countries that 509 cases occurred in MSM, and 218 cases were HIV positive^6^. Perez et al. analyzed 27 cases in Portugal, of which 26 were MSM and 14 had concurrent HIV infection^3^. As in the reported case, the infection mainly affected the MSM group, and many of the cases had previously been infected by HIV^3^ ^,^ ^6^ ^,^ ^7^. However, even in patients with HIV, the course of the disease was benign, unlike what was observed in our patient, who progressed in severity due to the morbidity associated with the number of mucocutaneous lesions.

The patient was hospitalized for 14 days. The primary reasons for hospitalization were pain management, particularly severe anorectal pain, pharyngitis that limited oral intake, and infection control. Subsequently, the patient developed urinary flow obstruction, respiratory failure, acute kidney injury, and multiple organ dysfunctions. Thornhill et al. reported a hospitalization rate of 13% in a sample of 528 cases diagnosed between April 27 and June 24, 2022, in 16 countries. No deaths were reported^6^.

The estimated mortality rate ranges from 1% to 11%. In a systematic review published this year, Bunge et al. calculated that the case fatality rate was 8.7%^5^. By July 27, 2022, the Brazilian Ministry of Health had reported 978 cases in Brazil; however, no deaths had been reported to date. This case represents the first death caused by the disease outside Africa during the current outbreak.

The multiple organ dysfunctions presented suggest that the most likely cause of death was sepsis, although there was no bacterial growth in several blood cultures and the patient was under broad-spectrum antibiotic therapy. The severely disseminated clinical presentation of the disease predisposed the patient to many conditions that made him susceptible to sepsis. Other causes of death can be hypothesized, including a visceral involvement of the disease. Considering the uniqueness of this case and the possible visceral involvement, the lack of a full autopsy and an accurate imaging examination can be considered major limitations of this report.

There are no records of contamination by healthcare professionals involved in patient care. However, Zachary and Shenoy described the risk of contamination among healthcare professionals involved in the medical care of previously endemic cases. Therefore, it is essential to understand the factors involved in the risk of contamination for these professionals to generate preventive information and post-exposure recommendations^8^.

On July 23, 2022, the World Health Organization (WHO) declared the escalating global monkeypox outbreak a public health emergency of international concern (PHEIC). Therefore, this atypical, severely disseminated clinical presentation of monkeypox virus infection points to the possibility of progression to severe disease in the current outbreak. Further studies are needed to better understand the factors involved in unfavorable clinical courses and their associated outcomes.

## References

[1] Guarner J, Del Rio C, Malani PN (2022). Monkeypox in 2022-What Clinicians Need to Know. JAMA Health Forum.

[2] Patrocinio-Jesus R, Peruzzu F (2022). Monkeypox Genital Lesions. N Engl J Med.

[3] Perez Duque M, Ribeiro S, Martins JV, Casaca P, Leite PP, Tavares M (2022). Ongoing monkeypox virus outbreak, Portugal, 29 April to 23 May
2022. Euro Surveill.

[4] Petersen E, Kantele A, Koopmans M, Asogun D, Yinka-Ogunleye A, Ihekweazu C (2019). Human Monkeypox: Epidemiologic and Clinical Characteristics,
Diagnosis, and Prevention. Infect Dis Clin North Am.

[5] Bunge EM, Hoet B, Chen L, Lienert F, Weidenthaler H, Baer LR (2022). The changing epidemiology of human monkeypox-A potential threat?
A systematic review. PLoS Negl Trop Dis.

[6] Thornhill JP, Barkati S, Walmsley S, Rockstroh J, Antinori A, Harrison LB (2022). Monkeypox Virus Infection in Humans across 16 Countries -
April-June 2022. N Engl J Med.

[7] Antinori A, Mazzotta V, Vita S, Carletti F, Tacconi D, Lapini LE (2022). INMI Monkeypox Group. Epidemiological, clinical and virological
characteristics of four cases of monkeypox support transmission through
sexual contact, Italy, May 2022. Euro Surveill.

[8] Zachary KC, Shenoy ES (2022). Monkeypox transmission following exposure in healthcare
facilities in nonendemic settings: Low risk but limited
literature. Infect Control Hosp Epidemiol.

